# Formulation and Ex Vivo Evaluation of Ivermectin Within Different Nano-Drug Delivery Vehicles for Transdermal Drug Delivery

**DOI:** 10.3390/pharmaceutics16111466

**Published:** 2024-11-18

**Authors:** Eunice Maureen Steenekamp, Wilna Liebenberg, Hendrik J. R. Lemmer, Minja Gerber

**Affiliations:** Centre of Excellence for Pharmaceutical Sciences (Pharmacen™), North-West University, Potchefstroom 2531, South Africa; eunicesteenekamp13@gmail.com (E.M.S.); wilna.liebenberg@nwu.ac.za (W.L.); righard.lemmer@nwu.ac.za (H.J.R.L.)

**Keywords:** ivermectin, nano-emulgel, nano-emulsion, nanoparticles, nanotechnology, transdermal drug delivery, topical drug delivery, dermal cytotoxicity, HaCaT, BJ-5ta

## Abstract

**Background/Objectives:** Ivermectin gained widespread attention as the “miracle drug” during the coronavirus disease 2019 (COVID-19) pandemic. Its inclusion in the 21st World Health Organization (WHO) List of Essential Medicines is attributed to its targeted anti-helminthic response, high efficacy, cost-effectiveness and favorable safety profile. Since the late 2000s, this bio-inspired active pharmaceutical ingredient (API) gained renewed interest for its diverse therapeutic capabilities. However, producing ivermectin formulations does remain challenging due to its poor water solubility, resulting in low bioavailability after oral administration. Therefore, the transdermal drug delivery of ivermectin was considered to overcome these challenges, which are observed after oral administration. **Methods:** Ivermectin was incorporated in a nano-emulsion, nano-emulgel and a colloidal suspension as ivermectin-loaded nanoparticles. The nano-drug delivery vehicles were optimized, characterized and evaluated through in vitro membrane release studies, ex vivo skin diffusion studies and tape-stripping to determine whether ivermectin was successfully released from its vehicle and delivered transdermally and/or topically throughout the skin. This study concluded with cytotoxicity tests using the methyl thiazolyl tetrazolium (MTT) and neutral red (NR) assays on both human immortalized epidermal keratinocytes (HaCaT) and human immortalized dermal fibroblasts (BJ-5ta). **Results:** Ivermectin was successfully released from each vehicle, delivered transdermally and topically throughout the skin and demonstrated little to no cytotoxicity at concentrations that diffused through the skin. **Conclusions:** The type of nano-drug delivery vehicle used to incorporate ivermectin influences its delivery both topically and transdermally, highlighting the dynamic equilibrium between the vehicle, the API and the skin.

## 1. Introduction

Ivermectin has been regarded as a “miracle drug” during the COVID-19 (coronavirus disease 2019) pandemic, but its significance in the medical and veterinary fields dates back to the 1970s, when it was first discovered in the fermentation broth of the soil bacterium, *Streptomyces avermitilis*, by Satoshi Õmura [1]. Ivermectin was derived from the fermentation process of *Streptomyces avermitilis* and underwent chemical modification to produce ivermectin (22,23-dihydroavermectin B1) and was, therefore, classified as a semi-synthetic avermectin [2,3]. Structurally, ivermectin consists of a macrocyclic lactone ring with various sugar moieties, contributing to its low water solubility and high lipophilicity [4].

Ivermectin acts by selectively binding to glutamate-gated ion channels and increasing the cell membrane’s permeability to specific ions. This causes hyperpolarization of the cells and subsequent paralysis of invertebrates [5]. Annually, almost 250 million people use ivermectin to combat various parasitic diseases, including scabies, pediculosis, or head lice, papulopustular rosacea, onchocerciasis, strongyloidiasis, ascariasis, cutaneous larva migrans and filariasis [6,7,8,9,10]. Besides its antiparasitic activity, studies have demonstrated its antibacterial and antiviral effectiveness [11,12,13]. Although ivermectin has been extensively researched for its potential effectiveness against the severe acute respiratory syndrome coronavirus 2 (SARS-CoV-2 virus), recent studies concluded that it did not significantly affect COVID-19 patient outcomes [14,15]. In more recent studies, evidence supporting the anticarcinogenic activity of ivermectin, as well as its anti-plasmodial activity for possible malaria intervention, has been highlighted [16,17,18].

Although ivermectin has been used and studied for the treatment of various infections, it is classified under the Biopharmaceutical Classification System (BCS) as a Class II drug. This classification suggests that ivermectin has high membrane permeability but low aqueous solubility, indicating that its bioavailability might be limited by its dissolution rate [19,20,21]. No serious adverse events have been reported in patients treated with standard therapeutic dosages of ivermectin, although moderate gastrointestinal side effects such as nausea, vomiting, diarrhea and stomach pain may occur [6,7]. The poor water solubility associated with ivermectin proves challenging with regard to the development of different dosage forms as low bioavailability is observed after oral administration [13].

The transdermal delivery of ivermectin holds promise in overcoming these challenges as the high lipophilicity of ivermectin may prove advantageous in producing transdermal drug delivery vehicles, resulting in optimum transdermal delivery of the API [17,22]. This route avoids hepatic first-pass metabolism, reducing side effects associated with oral administration and boosting bioavailability [23].

While extensive research has been conducted on nano-formulations combined with ivermectin, studies have primarily focused on specific applications. For instance, lipid nanocapsules have been investigated for their insecticidal activity treating *Pediculus humanus capitis* (the authors stated that these formulations might be suitable for topical administration, since their pH was higher than 6) [24]; subcutaneous injection of poly (lactic-co-glycolic acid) nanoparticles have been studied in a rodent model for therapeutic efficacy against *Brugia malayi* [25]; and polyanhydride nanoparticles have been assessed in cell culture studies for potential use against *Brugia malayi* filarial worms [26]. Lipid nanoparticles, including nanostructured lipid carriers and solid lipid nanoparticles combined with methoprene, were formulated and characterized for future veterinary applications [27], while liposomal systems were explored in antiviral cell culture studies [28]. Lipid nanocapsules were inspected for antiparasitic veterinary applications after injecting Wistar rats subcutaneously [29], histopathology and in vivo wound healing activity was explored in albino Wistar rats using a nanoliposomal gel (the authors concluded that wound healing was effective due to the deeper skin permeation of ivermectin) [30] and mesoporous silica and polymeric nanocapsules were examined to determine their in vitro release, solubility and drug loading capabilities [31]. Additional examples include nanofibers combined with ciprofloxacin for testing antimicrobial efficacy and wound healing in both in vitro and in ovo studies [32], and multi-walled carbon nanotube investigated in Wistar rats for effects on locomotor activity and neuropathic pain [33]. Other studies have compared solid lipid nanoparticles with an ivermectin suspension to assess the ex vivo diffusion using excised rat abdominal skin; the solid lipid nanoparticles demonstrated a higher flux than the suspension, although no tape stripping was conducted to evaluate topical drug delivery [34]. In a separate study, nanocrystals were used to explore the ex vivo diffusion through adult pig ear skin; the ivermectin solution showed higher drug diffusion through the skin compared to the nanocrystals, while the nanocrystals improved the skin retention of ivermectin [35]. However, at the time of preparing this research paper, no studies have focused on developing ivermectin-loaded nano-emulsions, ivermectin-loaded nano-emulgels or colloidal suspensions with ivermectin-loaded nanoparticles specifically for ex vivo transdermal and topical drug delivery. Furthermore, in this study, a natural oil with a favorable safety profile was incorporated into the nano-drug delivery vehicles to serve as a penetration enhancer.

When comparing the physicochemical properties of ivermectin (molecular mass of 875.10 Da (22,23-dihydroavermectin B1a) and 861.07 Da (22,23-dihydroavermectin B1b), aqueous solubility of 0.004 mg/mL, log P of 3.2 and melting point of 157 °C [3,36,37,38,39,40]) to the optimal physicochemical properties for skin permeation (molecular mass of less than 500 Da, aqueous solubility of 1.000 mg/mL or more, log P between 1.0 and 3.0 and melting point of less than 200 °C) suggested by Naik et al. [22], it is observed that only the melting point of ivermectin meets the optimal criteria for transdermal and topical drug delivery. Additionally, due to its lipophilic nature, ivermectin is expected to preferentially remain in the lipophilic layer of the skin, the stratum corneum. Therefore, to enhance topical and especially transdermal drug delivery, nano-drug delivery vehicles can be used to optimize drug delivery into and through the skin. Furthermore, a penetration enhancer can be added to mitigate the skin’s barrier function, promoting deeper penetration [41].

Natural oils, comprising both saturated and unsaturated fatty acids (UFAs), act as chemical penetration enhancers by disrupting the ordered structure (lipid bilayer) of the stratum corneum [42]. During this study, evening primrose oil (EPO) was incorporated as natural oil to serve as both chemical penetration enhancer and oil phase during formulation. EPO contains ~70–74% linoleic acid, 8–10% ɣ-linolenic acid, ~7% oleic acid, ~6% palmitic acid and up to 3% of other fatty acids [43]. Linoleic acid, an example of an UFA with a C_18_ chain length [42], is known for its optimal penetration-enhancing effects, followed closely by oleic acid and linolenic acid [44]. These fatty acids are natural components of the skin [42] and are therefore likely to be gentler on the skin compared to synthetic chemical penetration enhancers [45,46].

Although ivermectin lacks the optimal physicochemical properties for skin permeation, this study aimed to investigate its potential for transdermal and/or topical delivery using different nano-drug delivery vehicles (namely a nano-emulsion, nano-emulgel and a colloidal suspension with ivermectin-loaded nanoparticles) and incorporating a natural oil, EPO, as a chemical penetration enhancer.

## 2. Materials and Methods

### 2.1. Materials

Ivermectin was purchased from DB Fine Chemicals (Sandton, South Africa). Both the lipophilic surfactant (Span^®^ 60) and hydrophilic surfactant (Tween^®^ 80) utilized during the formulation of the nano-emulsions and nano-emulgels, along with the excipients used to produce the nanoparticles, which included polycaprolactone (PCL), polyvinyl alcohol (PVA) and dichloromethane (DCM), were purchased from Sigma-Aldrich (Johannesburg, South Africa). Carbopol^®^ Ultrez 20, incorporated in the nano-emulgels as a gelling agent, was sourced from Lubrizol (Durban, South Africa). EPO was sourced from Scatter Oils (Johannesburg, South Africa). The phosphate-buffered solution (PBS; pH 7.4) was prepared with potassium dihydrogen phosphate (KH_2_PO_4_) and sodium hydroxide (NaOH), both obtained from Sigma-Aldrich (Johannesburg, South Africa). The Direct Pure^®^ 40 Ultrapure laboratory water purification system (Merck-Millipore, Midrand, South Africa) was used to supply ultrapure (UP) water throughout the study. During high-performance liquid chromatography (HPLC) analysis, chromatography-grade methanol together with analytical-grade formic acid were used, both sourced from Associated Chemical Enterprises (ACE) (Johannesburg, South Africa). Dow Corning^®^ high vacuum grease, Whatman^®^ filter paper and Parafilm^®^ were utilized during in vitro membrane release and ex vivo skin diffusion studies and obtained from Separations (Randburg, South Africa).

Materials used in the cytotoxicity studies included Dulbecco’s Modified Eagle’s Medium (DMEM) and Trypan Blue solution (0.4%) (both from HyClone™, Separations, Johannesburg, South Africa), 1% non-essential amino acids (NEAAs), Trypsin-Versene^®^ ethylenediaminetetraacetic acid (EDTA) and 1% penicillin/streptomycin (pen/strep; 10,000 U/mL) (all from Lonza, Whitehead Scientific (Pty) Ltd., Cape Town, South Africa), 10% fetal bovine serum (FBS; Gibco, Thermo Fisher Scientific, Johannesburg, South Africa) and Hygromycin^®^ (Sigma-Aldrich).

### 2.2. Quantification of Ivermectin

An HPLC analytical method for the detection of ivermectin was successfully developed, optimized and validated at controlled chromatographic conditions. The Shimadzu^®^ Nexera-I LC-2040C 3D HPLC (Shimadzu South Africa, Roodepoort, South Africa), equipped with a quaternary pump, column heater, diode-array detection (DAD) detector and autosampler injector mechanism, connected to a LabSolutions Rev. A.10.03 acquisition and analysis software, were used to analyze the chromatograms. The room temperature of the laboratory in which the apparatus was stationed maintained a temperature of ±23 °C. A Venusil XBP C_18_(2) reverse phase column (150 × 4.6 mm) (Agela Technologies, Newark, Germany) with a particle size of 5 μm was utilized. The mobile phase was made up of two phases (A and B); phase A constituting UP water and phase B, consisting of methanol. Analytical-grade formic acid (0.1% *v*/*v*) was added to both phases, respectively. The isocratic elution method was used with 12% of mobile phase A and 87% of mobile phase B. The injection volume was set at 5 μL and the flow rate and UV detector were predetermined at 0.4 mL/min and 243 nm, respectively, for the detection of ivermectin. Ivermectin had a retention time of 3.1 min with a total run time of 5.0 min. The limit of detection (LOD) and limit of quantification (LOQ) of ivermectin was calculated as 0.316 and 0.500 μg/mL, respectively.

### 2.3. Preparation of a Standard Solution

A standard solution was prepared for each analysis. A linear regression curve was obtained by preparing and injecting this standard solution and its corresponding serial dilutions into the HPLC. Each standard solution was prepared by diluting 25 mg of ivermectin with 100 mL of chromatography-grade ethanol within a volumetric flask. Two-fold serial dilutions were performed to produce the required dilutions for the applicable concentration range. Each dilution was obtained by transferring a certain amount of the proceeding dilution into a test tube and dissolving it in the same amount of ethanol. For example, Dilution 1 was prepared by transferring 5 mL of the standard solution into a test tube and then dissolving it in 5 mL of ethanol. Dilution 2 was obtained by transferring 5 mL of the standard solution into a test tube and then dissolving it in 5 mL of ethanol, and so forth. Each sample was filtered into a marked HPLC vial for analysis. Finally, each sample was injected, and a linear regression curve was produced with an R^2^ value of ~1.00.

### 2.4. Physicochemical Properties of Ivermectin

#### 2.4.1. Solubility of Ivermectin in Evening Primrose Oil

Firstly, four polytops were marked and filled with 5 mL EPO each. The polytops were submerged in a preheated (~32 °C—imitating the epidermal surface temperature) water bath (Grant^®^ JB series water bath, Grant Industries, Cambridgeshire, UK) with a Variomag^®^ magnetic stirring plate (Variomag, Daytona Beach, FL, USA) [47]. Three of the polytops were oversaturated with ivermectin, while the fourth polytop served as a control for the experiment and contained only EPO. Magnetic stirrers were added to each polytop and left to stir for 24 h. Afterwards, the three polytops (oversaturated with ivermectin) were removed from the water bath and 1 mL was extracted from each polytop, filtered and transferred into three respective 100 mL volumetric flasks and diluted to volume with ethanol (chromatography grade). These three solutions were sonicated in an ultrasonic bath (Elma Transonic EL540, Singen am Hohentwiel, Germany) to ensure adequate dissolution. Finally, approximately 1 mL of the solutions was filtered into marked HPLC vials for analysis in duplicate. The fourth marked polytop contained only EPO and was prepared following the same method and the sample was also analyzed in duplicate.

#### 2.4.2. Solubility of Ivermectin in PBS (pH 7.4)

The solubility of ivermectin in PBS (pH 7.4) was determined by performing a solubility study following the same method as mentioned for the solubility in EPO. The exception being that PBS (pH 7.4) was used instead of EPO. The HPLC analysis of each sample occurred in duplicate.

#### 2.4.3. Solubility of Ivermectin in *n*-Octanol

To establish whether ivermectin exhibits more lipophilic properties, a solubility study was performed following the same method as mentioned for solubility in EPO. The exception being that *n*-octanol was used instead of EPO.

#### 2.4.4. Octanol-Buffer Distribution Coefficient of Ivermectin

Equal amounts (10 mL) of *n*-octanol and PBS (pH 7.4) were placed in a beaker and stirred for 24 h with a magnetic stirrer, ensuring co-saturation of the two phases [48]. Thereafter, the co-saturated phases were placed in a separation funnel and allowed to separate. After the mixture separated with the top phase consisting of *n*-octanol and the bottom phase of PBS (pH 7.4), both phases were emptied into separate beakers and marked accordingly. From the solubility test results, it was established that 163 mg of ivermectin should be added to 1 mL of the pre-saturated PBS (pH 7.4). Subsequently, 326 mg ivermectin was added to three polytops (each containing a magnetic stirrer) filled with 2 mL of pre-saturated PBS (pH 7.4). The polytops were submerged in a Grant^®^ JB series water bath (Grant Industries, Cambridgeshire, UK) (pre-heated to ~32 °C) with a Variomag^®^ magnetic stirring plate (Variomag, Daytona Beach, FL, USA) to stir for 45 min. Pre-saturated *n*-octanol (2 mL) was added to each polytop (containing PBS (pH 7.4) and ivermectin). These samples were then left to stir for an additional 2 h. Afterwards, the three samples were removed from the water bath and transferred into marked Falcon^®^ tubes for centrifugation (Haraeus Multifuge 1 L-R centrifuge, Thermo Fisher Scientific, Johannesburg, South Africa) at 11,000 rpm (15,557× *g*) for 30 min. A volume of 1 mL of supernatant (*n*-octanol) from each sample was diluted to 100 mL with ethanol and sonicated for 5 min. Approximately, 1 mL of each sample was filtered into marked HPLC vials for analysis in duplicate. The bottom layer of the Falcon^®^ tube consisted of the PBS (pH 7.4). Using a micropipette, 1 mL was extracted and diluted with ethanol to 100 mL and sonicated for 5 min. The PBS (pH 7.4) samples were filtered into three marked HPLC vials for analysis in duplicate. The log D (octanol-buffer distribution coefficient) was calculated by determining the concentration ratio of ivermectin that was detected in both *n*-octanol and PBS (pH 7.4). Equation (1) was used to determine the log D value of ivermectin [48].

(1) $$\mathrm{Log}\;D=\frac{\mathrm{Concentration}\;\mathrm{of}\;\mathrm{ivermectin}\;\mathrm{in}\;n\text{-}\mathrm{octanol}}{\mathrm{Concentration}\;\mathrm{of}\;\mathrm{ivermectin}\;\mathrm{in}\;\mathrm{PBS}\;(\mathrm{pH}\;7.4)\;}$$

### 2.5. Formulation of Nano-Drug Delivery Systems Containing Ivermectin

#### 2.5.1. Formulation of a Nano-Emulsion Containing Ivermectin

Four different nano-emulsion formulas (see Table S1) were selected, developed and characterized (see Table S2) to establish the best nano-emulsion formula for the delivery of ivermectin. The four o/w nano-emulsions were formulated with each containing EPO, UP water, ivermectin (2%) and different ratios of the surfactants, Span^®^ 60 and Tween^®^ 80. Ivermectin and Span^®^ 60 were added to EPO (oil phase) and stirred on a magnetic hot plate at ~80 °C until fully dissolved. Tween^®^ 80 was added to UP water (water phase) and stirred on a magnetic hot plate at ~80 °C. Once both phases reached the same temperature and the API and surfactants had fully dissolved, the oil phase was added dropwise into the continuously stirred aqueous phase. This formed a coarse emulsion, which was left to stir for an additional 5 min. Once cooled to room temperature (25 °C), each course emulsion was transferred to the ultrasonicator (Model UP200St, Hielscher Ultrasonics, Teltow, Germany) and sonicated for ±3 min to produce a nano-emulsion.

Among the four preformulated nano-emulsions (Table S2), **NE3** was identified as the superlative nano-emulsion, exhibiting the most favorable properties (smallest droplet size, good polydispersity index (PDI) indicating a homogenously dispersed formulation, and the highest zeta-potential, suggesting long-term stability) and named **NE** (Table 1). The placebo counterpart to **NE**, named **PNE**, was also formulated, serving as a control group throughout this study [48]. Table 1 displays the formulas used to produce the applicable nano-drug delivery vehicles.

#### 2.5.2. Formulation of a Nano-Emulgel Containing Ivermectin

The formula of the optimized **NE** was used to formulate four nano-emulgels (see Table S4) by adding different concentrations (*w*/*w*) of Carbopol^®^ Ultrez 20. The preparation of the oil phase was identical to that of the **NE**, while Carbopol^®^ Ultrez 20 was added to the aqueous phase after Tween^®^ 80 was completely dissolved in the UP water. Whilst the aqueous phase underwent homogenizing, the oil phase was added dropwise to the aqueous phase and homogenized for 10 min to form the emulgel. The emulgel was then transferred to the ultrasonicator (Model UP200St, Hielscher Ultrasonics, Teltow, Germany) and sonicated for approximately 2 min to form the nano-emulgel [48]. Once cooled to room temperature (25 °C), the pHs of the nano-emulgels were adjusted with NaOH (optimal viscosity is reached at pH 6–7). The respective placebo of the optimized nano-emulgel (**NEG**) (see Table S5 for the characterization results of the four preformulated NEGs) was also produced and named **PNEG** [49].

#### 2.5.3. Formulation of a Colloidal Suspension Containing Ivermectin-Loaded Nanoparticles

To prepare the colloidal suspension, ivermectin-loaded nanoparticles were produced by means of the emulsion-solvent evaporation method [50]. The aqueous phase was prepared by stirring PVA in UP water on a magnetic hot plate at ~80 °C for 1 h, or until the PVA was sufficiently dissolved. The organic phase was prepared by dissolving equal quantities of ivermectin and PCL in DCM and stirring it for 20 min, until dissolved. The aqueous phase was allowed to cool on ice before adding the organic phase dropwise to the aqueous phase while sonicating. This produced an emulsion with a milky-white appearance. The emulsion was transferred to a rotary evaporator (Büchi^®^ Rotavapor^®^ RII, Labotec, Midrand, South Africa) where the organic phase was evaporated under controlled temperature and pressure. The appearance of the emulsion changed to an opaque-watery consistency. The emulsion was transferred into centrifuge tubes for centrifugation (Haraeus Multifuge 1 L-R centrifuge, Thermo Fisher Scientific, Johannesburg, South Africa) for 30 min at 4 °C and 11,000 rpm (15,557× *g*). Afterwards, the nanoparticle pellet was rinsed with UP water to remove any residual organic phase. The remaining pellet was re-dispersed in UP water and placed in an ultrasonic bath for 15 min. Sucrose was added as a cryoprotectant in a ratio of one part sucrose to two parts nanoparticles. The tubes were stored in a −80 °C freezer overnight before being freeze-dried for 72 h. Finally, the dry nanoparticles were stored in a desiccator to remove excess moisture and to keep the particles dry. The dry nanoparticles were labelled **NPs**. After enough ivermectin-loaded **NPs** were formulated, the correct quantity of **NPs** was weighed and suspended in EPO to produce a colloidal suspension, labelled **CS** [48,50]. Due to sedimentation and a paste-like consistency observed at a 2.00% (*w*/*v*) ivermectin concentrations, **CS** was formulated at a reduced concentration of 0.35% (*w*/*v*).

### 2.6. Characterization of Nano-Drug Delivery Vehicles

#### 2.6.1. Visual Examination

All visual examinations were performed directly after each nano-drug delivery vehicle was successfully formulated to ensure no visual instabilities were present, such as creaming, flocculation and sedimentation. Visual examinations took place on days 1, 3 and 7.

#### 2.6.2. pH

A Mettler Toledo^®^ pH meter (Mettler Toledo, Columbus, OH, USA) was used to measure the pH of each nano-drug delivery vehicle. Before measuring the pH, the system was calibrated at a pH of 4, 7 and 10. The Mettler Toledo^®^ InLab^®^410 electrode was placed into each nano-drug delivery vehicle and the pH was measured in triplicate [48].

#### 2.6.3. Viscosity

The viscosity of all the nano-emulsions and nano-emulgels was measured using a Brookfield viscometer DV2T LV (Brookfield Engineering, Middleborough, MA, USA) attached to a thermostatic water bath. Each nano-drug delivery vehicle was placed in a preheated water bath at 25 °C for ±60 min before measuring took place. A T-B spindle was attached to the viscometer and the rotation speed was set at 100 rpm for the nano-emulsions’ measurements. For the nano-emulgels, a T-F spindle was selected, and the rotation speed was also set at 100 rpm. Viscosity data of each nano-drug delivery vehicle were obtained at set multipoint measurements within time intervals of 2 min for 10 min. The average viscosity (cP) and torque (%) of each nano-drug delivery vehicle were determined.

#### 2.6.4. Droplet/Particle Size and Polydispersity Index

To determine the droplet/particle size and PDI of all the nano-drug delivery vehicles, samples were prepared as follows: one drop of each drug delivery vehicle was transferred into its respective polytop and diluted with 10 mL UP water. Each polytop was placed in an ultrasonic bath for 10 min to ensure adequate dissolution. Afterwards, a syringe was used to extract 2 mL from each polytop and to fill a clear disposable low-volume cell (Zen0112). A Zetasizer Nano ZS (Malvern Instruments, Malvern, UK) was used to measure the mean droplet/particle size and PDI of each sample in triplicate. It is of note that the ivermectin-loaded **NP** size was determined before and after it underwent freeze-drying.

#### 2.6.5. Zeta-Potential

A Malvern Zetasizer Nano ZS (Malvern Instruments, Malvern, UK) measured the zeta-potential of each nano-drug delivery vehicle in triplicate. The same method was used to measure the droplet/particle size and PDI was used; however, clear disposable capillary zeta-cells were used containing 2 mL of each sample.

#### 2.6.6. Entrapment Efficiency

On determining the entrapment efficiency (EE%), approximately 15 mL of each sample was centrifuged with a Beckman Coulter Optima XPN-100 Ultracentrifuge (Centurion, South Africa) at 63.9× *g* (23,000 rpm) for 15 min at 23 °C. Subsequently, the oil and aqueous phases were clearly separated. The oil phase (200 µL) of each nano-emulsion sample was extracted and transferred to a 5 mL volumetric flask, where it was diluted to volume with ethanol and sonicated for 5 min. The samples were filtered into marked HPLC flasks for HPLC analysis in duplicate. A standard curve was also prepared and analyzed according to the same method as previously described [48]. Equation (2) was utilized to determine the entrapment efficiency for each nano-emulsion, where W_N_ is the entrapped amount API, W_T_ is the total amount of API and W_F_ is the unentrapped amount of API [51].

(2) $$\mathrm{Entrapment}\;\mathrm{efficiency}=\frac{W_{N}}{W_{T}}\text{×}100$$

W_T_ = W_F_ + W_N_ (3)

#### 2.6.7. Encapsulation Efficiency

After the ivermectin-loaded **NPs** were formulated, the concentration of ivermectin encapsulated within the **NPs** was determined. HPLC analysis was utilized to quantify the concentration of ivermectin retrieved from the produced **NPs**. One batch of formulated **NPs** weighed approximately 40 mg and, theoretically, contained 30 mg of ivermectin. One batch of produced **NPs** was transferred to a 100 mL volumetric flask and diluted with 100 mL ethanol. The solution was filtered into an HPLC vial and analyzed in duplicate. The data were plotted against a standard linear regression curve obtained from a standard solution. The encapsulation efficiency for the ivermectin-loaded **NPs** was determined as 52.44% using Equation (4) [48,52].

(4) $$\mathrm{Encapsulation}\;\mathrm{efficiency}=\frac{\mathrm{Actual}\;\mathrm{drug}\;\mathrm{content}}{\mathrm{Theoretical}\;\mathrm{drug}\;\mathrm{content}\;}\text{×}100$$

#### 2.6.8. Morphology

Scanning electron microscopy (SEM) was utilized to evaluate the morphology (shape, size and texture) of the formulated **NP**. The **NP** was attached to a small piece of carbon tape for analysis after being stored in the desiccator for three days. The tape was set on a metal mount and sputter-coated with gold-palladium film (Eiko Engineering Ion Coater IB-2, Tokyo, Japan). A 10 kV accelerated voltage was programmed on the FEI Quanta 200 FEG SEM and the X-Max 20 EDS system recorded the micrographs (Hillsboro, OR, USA).

#### 2.6.9. X-Ray Powder Diffraction Analysis

X-ray powder diffraction (XRPD) patterns were generated at an ambient temperature with a PANalytical Empyrean diffractometer (PANalytical, Almelo, The Netherlands) equipped with a PIXcel3D detector. Each powder was distributed evenly on a zero-background sample holder and the analysis conditions were set as follows: target: Cu; voltage: 45 kV; current: 40 mA; wavelength (λ): 1.5406 Å, and step size: 0.01°.

### 2.7. In Vitro Membrane Release Studies

The receptor phase mixture (PBS (pH 7.4) and 30% ethanol) [52,53], each nano-drug delivery vehicle containing ivermectin and its respective placebo were prepared beforehand. One hour before the in vitro membrane release study commenced, the prepared receptor phase mixture was immersed in a preheated (~37 °C) water bath (Grant^®^ JB series water bath, Grant Industries, Cambridgeshire, UK) to simulate the temperature of human blood. Additionally, each nano-drug delivery vehicle containing ivermectin and its respective placebo was immersed into a water bath, set at a temperature of ~32 °C, imitating the temperature of the skin surface. Prior to HPLC analysis of each in vitro membrane release study, a standard solution and serial dilutions thereof were prepared and injected into the HPLC to obtain a linear regression curve for analysis. In vitro membrane release studies were performed with each nano-drug delivery vehicle (**NE**, **NEG** and **CS**) to determine whether ivermectin was released effectively from the different formulated drug delivery vehicles from the donor compartment, through the membrane, into the receptor compartment of the Franz diffusion cells (PermeGear, Hellertown, PA, USA). A polyvinylidene fluoride (PVDF) synthetic membrane (Pall^®^ Life Sciences, Port Washington, WI, USA) with a pore size of 45 μm and diameter of 25 mm was placed between the donor and receptor phases of the Franz diffusion cell. Dow Corning^®^ high vacuum grease was applied to both compartments of the Franz cell (the top part of the receptor compartment and the bottom part of the donor compartment), facilitating the attachment of the two compartments. A small magnetic stirring rod was placed inside each receptor compartment while maintaining the receptor compartments in an upright and stable position. The PVDF membrane was meticulously placed on top of the receptor compartment. The donor compartment was placed on top of the receptor compartment and vacuum grease was applied to the sides/rims, this time to seal the cell. A horseshoe clamp was used to fasten the Franz diffusion cell, securing the intact cell and preventing any leakage during the study. A volume of 2 mL of the preheated receptor mixture (~37 °C) was injected into the receptor compartments of the Franz cells. The compartment was carefully inspected to ensure no air bubbles were present. The donor compartments (*n* = 10) were filled with a volume of 1 mL preheated (~32 °C) nano-drug delivery vehicles (**NE**, **NEG** or **CS**) and two of the donor compartments were filled with the 1 mL preheated (~32 °C) respective placebo (either **PNE**, **PNEG** or EPO) to function as control groups. Parafilm^®^ was used to seal the donor compartments. The 12 prepared Franz cells were placed in a Franz cell stand and immersed into the preheated Grant^®^ JB series water bath (Grant Industries, Cambridgeshire, UK) at ~37 °C equipped with a Variomag^®^ magnetic stirring plate (Variomag, Daytona Beach, FL, USA). This ensured continuous magnetic stirring within each receptor compartment. The process of extracting and refilling the receptor compartments was performed at 1-h intervals over a period of 6 h. The extracted receptor phase mixture was transferred to corresponding HPLC vials for HPLC analysis [48,54].

### 2.8. Skin Diffusion Studies

#### 2.8.1. Skin Preparation

Ex vivo skin diffusion studies were performed using human skin obtained from female Caucasian candidates who had undergone abdominoplasty procedures. Subjective selection of skin samples was based on voluntary donations from candidates undergoing the surgery. Ethics approval for the study was obtained from the North-West University Health Research Ethics Committee (NWU-HREC) prior to its commencement (Ethics no: NWU-00111-17-A1-18). Before initiating the ex vivo skin diffusion studies, all collected skin was dermatomed. A Dermatome™ (Zimmer Ltd., Staffordshire, UK) was pressed onto the skin at an angle of 45° to achieve a thickness of roughly 400 μm. The dermatomed skin samples were then transferred onto Whatman^®^ filter paper, wrapped in heavy-duty aluminum foil and tightly sealed in airtight plastic bags. The skin samples were then stored in a freezer at −20 °C until needed. All skin samples were meticulously examined for any potential flaws like tears, scars or stretch marks before being utilized in the ex vivo skin diffusion study [48,54,55].

#### 2.8.2. Ex Vivo Skin Diffusion Studies

The ex vivo skin diffusion studies were performed in accordance with the method employed during the in vitro membrane release studies; however, dermatomed skin samples (cut into small circles) were placed between the receptor and donor compartments of the Franz cells instead of the PVDF synthetic membranes. The dermatomed skin samples were positioned on the receptor phase with the stratum corneum facing upward (toward the donor phase) [55]. Ex vivo skin diffusion studies were performed accordingly with all three formulated nano-drug delivery vehicles (**NE**, **NEG** and **CS**) over 12 h with extraction and refilling of the receptor phases occurring 2-hourly. The collected samples were finally transferred to marked HPLC vials for analysis [48,54,55].

### 2.9. Tape Stripping

After skin diffusion was concluded, the Franz diffusion cell was dissembled followed by visual examination of the skin sample before being removed. The skin sample from each Franz cell was pinned to a small piece of Parafilm^®^ mounted on a solid surface. The skin sample was carefully dabbed to remove excess formulation. Approximately 16 pieces of 3M Scotch^®^ Magic™ tape were cut to fit over the skin diffusion area. The first strip of tape was pressed against the diffusion area and discarded, to avoid contamination. The remaining ±15 strips were pressed against the diffusion area and transferred to a correspondingly marked polytop, filled with 5 mL ethanol (extraction solution). Once tape stripping was completed, the remaining diffusion area of the skin sample from each Franz cell was cut into small pieces and transferred to a correspondingly marked polytop, also containing 5 mL ethanol. All marked polytops were then placed in a refrigerator at ~4 °C and left overnight (±12 h). Afterwards, the contents of the marked polytops were filtered into their marked HPLC vial for analysis. A standard solution and serial dilutions thereof were prepared and injected into the HPLC to obtain a linear regression curve against which to plot the data obtained from the tape stripping. Finally, the samples (containing tape strips and pieces of skin) were analyzed to determine the concentration of ivermectin present in the SCE (stratum corneum epidermis) and the ED (epidermis dermis), respectively [48,54,56,57].

### 2.10. Data Analysis

For both in vitro membrane release and ex vivo skin diffusion studies, the average cumulative amount per area released and diffused (expressed in µg/cm^2^) for ivermectin from each drug delivery vehicle was plotted against time. As a result, a linear regression curve was produced, and its slope served as a representation of the average rate or API flux (µg/cm^2^.h). The amount of API (%) released and/or diffused relative to the initial ivermectin concentration incorporated in each nano-drug delivery vehicle was also calculated.

Finally, the tape stripping method was used to calculate both the average and median concentration (expressed as μg/mL) of ivermectin that had been retrieved from the SCE and the ED.

### 2.11. In Vitro Cytotoxicity Studies

The epidermis consists predominantly of keratinocytes, which compromise approximately 95% of the skin cells [58]. Human immortalized epidermal keratinocytes (HaCaT) cells closely resemble normal human adult keratinocytes and can be utilized to investigate the dermal cytotoxicity of formulations, as mentioned by [59,60]. Fibroblasts are the main cell population found within the dermis and are conventionally associated with the production of extracellular matrix [61,62]. The BJ-5ta cell line is described as human skin fibroblast cells immortalized with human telomerase reverse transcriptase (hTERT) [63,64]. Therefore, during this study the extent of cytotoxicity in HaCaT and BJ-5ta cells inflicted by the exposure to the following treatments were investigated, namely **NE** (containing ivermectin); **PNE** (absent of ivermectin); **CS** (containing ivermectin) and API (ivermectin). The high viscosity displayed by the **NEG** posed limitations and was therefore not evaluated during this study.

#### 2.11.1. Cell Culturing Conditions

To maintain the HaCaT cells, they were cultured in a flask containing high-glucose DMEM supplemented with L-glutamine (2.0 mM), 1% NEAA, 1% pen/strep and 10% FBS. The BJ-5ta cells require storage in a flask containing Hygromycin^®^ (5 mg/mL). Afterwards, the flasks containing the cells were placed in an ESCO Cell Culture CO_2_ incubator (ESCO Technologies, Inc., St. Louis, MO, USA) at 37 °C with a 95% humidified atmosphere containing 5% CO_2_. Cells were inspected every 48 h to monitor any unfavorable bacterial growth, and the cell confluence was maintained at ±80–90%. Trypsinization [65] was performed by removing the growth media with a pipette and rinsing the flask twice with 10 mL of the preheated phosphate-buffered saline. Trypsin-Versene^®^ (3 mL) was added to detach the cells and a vented cap was placed on the flask to ensure even distribution of Trypsin-Versene^®^ over the flask’s surface. Cell viability was determined by preparing a counting solution of 25 μL of Trypan Blue solution (0.4%) and 15 μL phosphate-buffered saline and introducing it to the cell suspension (10 μL). The chamber of the hemocytometer was filled on both sides of the cover slip with 10 mL of the counting solution and viewed under a microscope. The cell suspension was diluted with DMEM to produce the seeding solution with a concentration of 75,000 cells/mL and 60,000 cells/mL for the HaCaT and BJ-5ta cells, respectively. Furthermore, the wells required a density of 15,000 cells/well for the HaCaT cells and 12,000 cells/well for the BJ-5ta cells. Thus, a volume of 200 μL of the seeding solution was transferred into each well with the multichannel pipette. The seeded well plates were incubated for 24 h at 37 °C, 5% CO_2_ and 95% humidity to allow the cells to recover, before the cells were treated with different concentrations of API, **NE**, **PNE** and **CS** for 12 h.

#### 2.11.2. Methyl Thiazolyl Tetrazolium (MTT) Assay

Both the HaCaT and BJ-5ta cell lines were treated identically. Once the 12 h treatment exposure period had lapsed, the plates were removed from the incubator and the MTT assay was performed. The treatment solution was aspirated from each well and rinsed twice with 100 μL of phosphate-buffered saline. The dead cells were killed after 15 min once 200 μL of Triton™ X-100 (0.2% in phosphate-buffered saline) had been added. The dimethyl sulfoxide (DMSO) blank wells were filled with 200 μL of preheated non-additive DMEM, while the untreated, treated and dead wells were filled with 180 μL of preheated non-additive DMEM. Subsequently, 20 μL of the MTT solution was added to the wells containing 180 μL of DMEM to obtain the required MTT concentration of 0.5 mg/mL [66]. Each well plate was then wrapped in aluminum foil and placed in the CO_2_ incubator for 4 h to allow the viable cells to metabolize the MTT. Once 4 h had elapsed, each well was aspirated again and filled with 200 μL of DMSO to dissolve the formed crystals. The plates were once again covered with aluminum foil and placed on a shaker for 1 h to ensure complete dissolution of the formazan crystals. A SpectraMax^®^ Paradigm^®^ multi-mode microplate reader (Molecular devices, San Jose, CA, USA) analyzed each plate. SoftMax^®^ Pro 6.2.1 software operated the microplate reader, and the absorbance was set to measure at a cell signal of 560 nm with a background signal of 630 nm [48,54].

#### 2.11.3. Neutral Red (NR) Assay

The NR assay also contained the same control groups as mentioned for the MTT assay, with the only exception being that DMSO was replaced with a solubilization blank. Once the treatment period had elapsed, all wells were aspirated and rinsed twice with 100 μL of phosphate-buffered saline. Thereafter, non-additive DMEM (200 μL) and 200 μL of the filtered 10.00% (*v*/*v*) NRS were added up to volume to the untreated, treated and dead cell control wells. The plates were covered with aluminum foil and incubated for 2 h. After 2 h of incubation, each well was aspirated and 100 μL of NR-fixative (1% calcium chloride (CaCl_2_)) in 0.5% formaldehyde) was added to the wells for fixation. Lastly, 150 μL of NR-solubilization solution (1% acetic acid in 50% ethanol) was added to the wells. The plates were then covered and placed on a shaker for 10 min before undergoing analysis with the same plate reader as mentioned for the MTT assay. For the NR assay, the absorbance was set to measure at a cell signal of 540 nm with a background signal of 690 nm [48,54].

### 2.12. Statistical Data Analysis

Statistical analysis of the data obtained for each nano-drug delivery vehicle during in vitro membrane release and ex vivo skin diffusion was conducted using Python 3.9 and the Statsmodels library. Data storage and handling from Excel were performed using the Pandas library, while vectorized computations were performed using the NumPy library. Figures were created using the Matplotlib and Seaborn libraries. With regard to the tape-stripping data obtained, ART ANOVA was performed using the ARTool package in R (version 4.1.3) [67,68,69,70,71,72].

When analyzing the data obtained from both the in vitro membrane release and ex vivo skin diffusion studies, a one-way ANOVA (analysis of variance) was performed to establish whether any statistical significance existed between the nano-drug delivery vehicles. Two-way ANOVA was used to evaluate statistical data obtained from the tape stripping tests with regard to the different nano-drug delivery vehicles. The *p*-value represents the likelihood of rejecting or failing to reject the null hypothesis (H_0_), which implies that there exists no appreciable difference between two groups with respect to a particular variable. The findings show statistical significance when the *p*-value is less than or equal to 0.05, indicating strong evidence against H_0_ [73].

## 3. Results and Discussion

### 3.1. Physicochemical Properties of Ivermectin

#### 3.1.1. Solubility of Ivermectin in EPO, PBS (pH 7.4) and *n*-Octanol

The solubility of ivermectin in EPO was determined as 9.049 ± 0.109 mg/mL. These results reflect the lipophilic nature of ivermectin as confirmed by its lipid solubility in *n*-octanol (162.778 ± 1.521 mg/mL). The solubility of ivermectin in PBS (pH 7.4) was determined as 0.054 ± 0.002 mg/mL, which further attests to its poor aqueous solubility (4.0 mg/L) as found in the literature [37].

#### 3.1.2. Octanol-Buffer Distribution Coefficient of Ivermectin

After determining the solubility of ivermectin in both PBS (pH 7.4) and *n*-octanol, the log D value of ivermectin was calculated as 3.686. The log D value (referring to the log P (octanol–water partition coefficient) at a specific pH, using a buffer instead of water) for ivermectin is similar to the log P value of ivermectin described in the literature as 3.200 [3,36]. With a log P value slightly higher than 3, ivermectin exhibits more lipophilic properties and, therefore, it can be predicted that ivermectin will permeate into the stratum corneum, as it has a higher affinity to lipophilic environments.

### 3.2. Characterization of Each Nano-Drug Delivery Vehicle

The characterization results of the nano-drug delivery vehicles are shown in Table 2.

#### 3.2.1. Visual Examination

All drug delivery vehicles showed no signs of physical instabilities, such as creaming, sedimentation and/or flocculation. The **NE** appeared homogenous, milky in color and had a watery consistency. Comparing the **NEG** to the **NE**, the **NEG** presented with a thicker consistency, as well as a glossy, gel-like appearance. This can be attributed to the addition of Carbopol^®^ Ultrez 20 in the nano-drug delivery vehicles. The **CS** containing ivermectin-loaded **NPs** presented with an opaque fluid and oily appearance. Initially, there was no sign of sedimentation, and it appeared stable, but after a few hours, some sedimentation occurred, since no stabilizer was added to the suspension.

#### 3.2.2. pH

All three nano-drug delivery vehicles (**NE**, **NEG** and **CS** in Table 2) measured a pH within the range of 3–9, which is well tolerated by the skin and, therefore, all vehicles were deemed safe for skin application [74].

#### 3.2.3. Viscosity

The **NE** demonstrated low viscosity, while the **NEG** had high viscosity. **NEs** face problems of low viscosity related to spreadability issues that can be rectified by the addition of a gelling agent. **NEGs** present with a higher viscosity due to their stable gel structure formed by cross-linking agents [75,76].

#### 3.2.4. Droplet/Particle Size and PDI

The ideal droplet size range for nano-drug delivery vehicles is between 20 and 200 nm; although, as mentioned in the literature, droplet sizes exceeding 150 nm are less likely to cross the skin’s barrier for transdermal/topical use [77]. Both the **NE** and **NEG** measured droplet sizes < 150 nm; even with the **NEG** displaying increased droplet sizes in comparison to the **NE**. The high degree of cross-linking associated with the addition of Carbopol^®^ Ultrez 20 is mostly the cause of the slightly enlarged droplets [78].

The particle size of the **NPs** was measured before and after freeze-drying as it was anticipated that the particle size of the **NPs** would increase after the addition of a cryoprotectant and undergoing freeze-drying [79]. Furthermore, it is worth noting that, even though **NPs** refer to particles within a size range of 1–100 nm, particles within a size range of 50–500 nm are also acceptable for the purpose of drug delivery [80]. Our anticipations were confirmed as the particle size measured before and after freeze-drying increased from 112.7 ± 4.2 nm to 173.8 ± 18.4 nm, respectively.

PDI values close to zero are preferred, as low PDI values (~0.1) are indicative of highly homogeneous formulations with a narrow droplet size distribution. PDI values near 1.0 can be classified as heterogeneously dispersed and unstable [81]. The **NE** measured the best PDI (~0.1), demonstrating the most uniform size distribution, followed by the **NEG** and finally, the **NPs** exhibited the poorest PDI, indicating that they had the largest size distribution and were more polydispersed than the **NE** and **NEG**.

#### 3.2.5. Zeta-Potential

Zeta-potential is directly related to the stability of a formulation. Generally, zeta-potential measurements of ≥±30 mV are electrically stabilized as coagulation or flocculation is less likely to occur within the dispersion [82,83]. All three nano-drug delivery vehicles exhibited adequate zeta-potential measurements, as seen in Table 2. The **NEG** presented with the lowest zeta-potential, followed by the **CS** and then the **NE**. The addition of Carbopol^®^ Ultrez 20 affects the surface charge of an emulsion’s droplets; subsequently, increasing the zeta-potential and stability of the drug delivery vehicle [78]. In conclusion, all three nano-drug delivery vehicles could be deemed stable, measuring zeta-potentials of <−30 mV.

#### 3.2.6. Entrapment Efficiency

It was established that the entrapment efficiency was poor for all four **NEs** prepared during pre-formulation, ranging between 17.87 and 18.36%. Based on the oil solubility of ivermectin provided in Section 3.1.1, the theoretical entrapment efficiency of ivermectin in EPO was calculated to be 9.049%, which indicated a low entrapment efficiency. However, the actual entrapment efficiency for ivermectin showed a significant improvement (2.029 times) over the theoretical value, suggesting that the surfactants contributed to enhancing the entrapment. In an attempt to further understand the nature of the poor results, the aqueous phase was also analyzed using the same method previously mentioned to determine the oil phase. Nearly the entire remaining fraction of ivermectin incorporated in the NE was found to be recoverable from the aqueous phase. A possible explanation for this could be that the finely dispersed oil phase (containing the API) did not separate sufficiently from the aqueous phase, as this is a common occurrence when formulating extremely small nano-drug delivery vehicles [51,84]. Typically, relatively high amounts of surfactants (10–15% *w*/*w*) are required to stabilize the high surface area of nano-sized droplets by adsorbing to the surface of the droplets, reducing surface tension and stabilizing the large interfacial surface [85]. In this study, Tween^®^ 80 was used as hydrophilic surfactant to stabilize the entrapped ivermectin droplets within the aqueous phase. This attests to the presence of ivermectin within the aqueous phase as no aggregation or precipitation was observed after centrifugation. The optimized **NE** containing 2% ivermectin was regarded as homogenous as reflected with the low PdI value (Table 2).

#### 3.2.7. Encapsulation Efficiency

Although lipophilic APIs, like ivermectin, are reported to produce high API encapsulation (~90%), it is important to note that a 100% API encapsulation is less likely. Factors that can lead to a decrease in encapsulation include loss of API due to the freeze-drying process and human error due to transfer between the multiple phases of formulation [86,87]. The encapsulation efficiency for the ivermectin-loaded **NPs** was determined as 52.44%.

#### 3.2.8. Morphology

**Figure 1:**
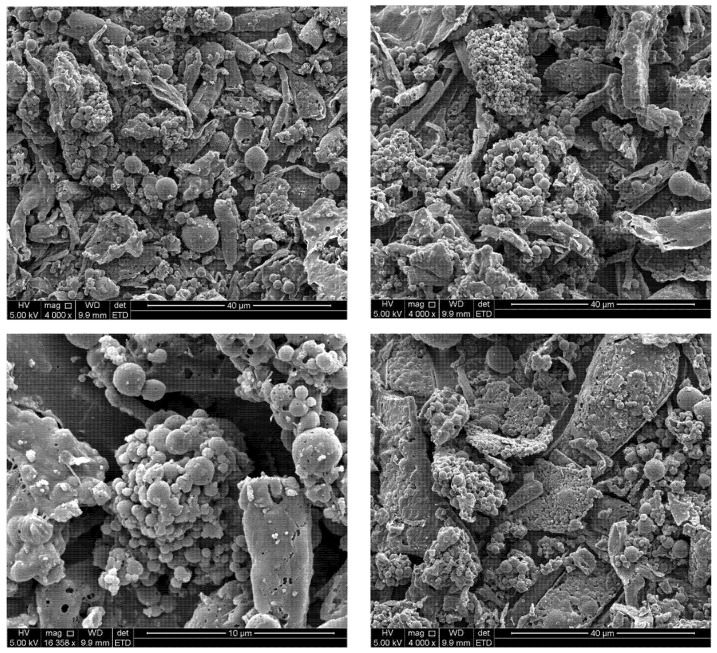
SEM micrographs of **NPs**.

Figure 1 illustrates the spherical **NPs** that fell within the nano-scale range, as recorded during SEM analysis.

#### 3.2.9. X-Ray Powder Diffraction Analysis

**Figure 2:**
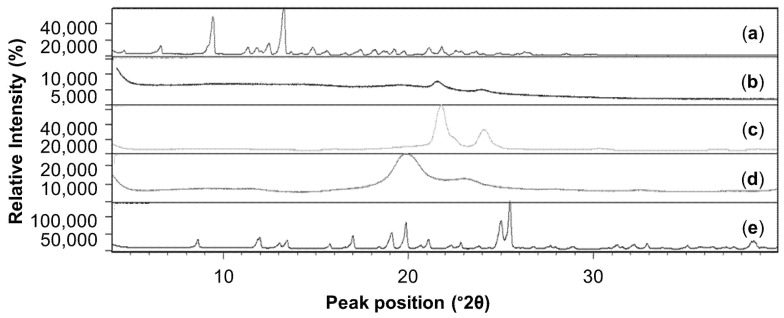
XRPD overlay for: (**a**) ivermectin, (**b**) ivermectin-loaded **NPs**, (**c**) PCL, (**d**) PVA and (**e**) sucrose.

When evaluating Figure 2, the ivermectin-loaded **NPs** indicated an amorphous nature as their diffraction pattern displayed no high-intensity diffraction peaks [88]. The diffractograms obtained for PCL and PVA also display no high-intensity diffraction peaks, while the diffractograms for ivermectin and sucrose exhibited multiple high-intensity diffraction peaks, indicative of a crystalline nature.

### 3.3. In Vitro Membrane Release Studies

**Figure 3:**
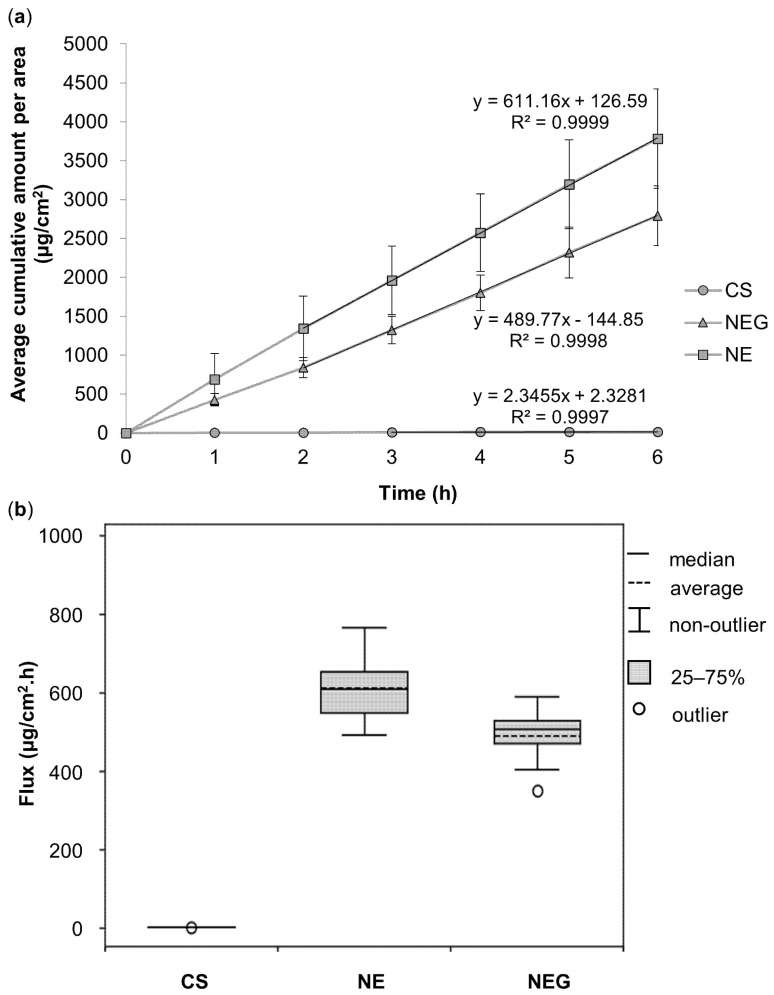
(**a**) Average cumulative amount of ivermectin released per area (μg/cm^2^) over 6 h during the in vitro membrane release studies from all the nano-drug delivery vehicles, and (**b**) boxplot displaying the average (dashed lines) and median (solid lines) flux (μg/cm^2^.h) of ivermectin for the nano-drug delivery vehicles during the in vitro membrane release studies over a period of 6 h.

The median flux of ivermectin will be discussed (indicated in Table 3), as it is regarded to be a more accurate method, since the outliers affect the median value less compared to the average values [89]. Additionally, Figure 3 was included to display the in vitro membrane release results obtained. As seen in Figure 3a, the cumulative amount of ivermectin released per area (μg/cm^2^) was plotted over 6 h in 1 h intervals to produce an average flux determined from 2 to 6 h for the **NE** and **NEG** and from 3 to 6 h for the **CS**. In vitro membrane release studies were performed to establish whether the API was released from the nano-drug delivery vehicles [42].

When referring to the median flux (μg/cm^2^.h) values in Table 3, **NE** measured the highest median flux, followed by **NEG** and lastly **CS** with the lowest median flux. When comparing the nano-drug delivery vehicles in terms of median %release, the same trend was observed.

The **CS** containing ivermectin-loaded **NPs** displayed the poorest drug release of all the nano-drug delivery vehicles. **NPs** are structurally stable as they possess a rigid matrix, allowing them to maintain their structural integrity for a longer period. This structural stability prolongs drug release time, explaining the slow release of ivermectin through the PVDF membrane [90]. Furthermore, it is also important to note that **CS** was formulated with an ivermectin content of 0.35% opposed to the 2.00% ivermectin in the other nano-drug delivery vehicles; therefore, it was expected that the **CS** would demonstrate lower flux values.

When comparing the flux of the **NE** to that of **NEG**, the **NE** demonstrated a higher median flux. The **NE** had a droplet size smaller than 100 nm and smaller in comparison to its gel-containing counterpart (**NEG**), producing an increased surface area and permitting a higher flux [91,92].

Furthermore, the viscosity of **NE** likely contributed to the improved API release of **NE** as it was significantly lower than that of **NEG**. The gel matrix produces a film that influences API release by retarding the flux of API [93,94,95]. A one-way ANOVA established that the nano-drug delivery vehicles all differed significantly from one another as the *p*-value was below a significance level of 0.05. Subsequently, the pair-wise Mann–Whitney U-tests revealed statistically significant differences between the mean flux of all three nano-drug delivery vehicles from one another (*p* < 0.05), with the most significant differences (*p* < 0.001) being between the **CS** and **NE**, as well as the **CS** and **NEG** groups. Therefore, it is unlikely that the data generated (between the statistically significant groups) occurred randomly but are rather indicative of a specific cause, which may be ascribed to the various properties of the different nano-drug delivery vehicles [96].

In conclusion, ivermectin was released from all three nano-drug delivery vehicles; thus, ex vivo skin diffusion studies could follow for each of them.

### 3.4. Ex Vivo Skin Diffusion Studies

**Figure 4:**
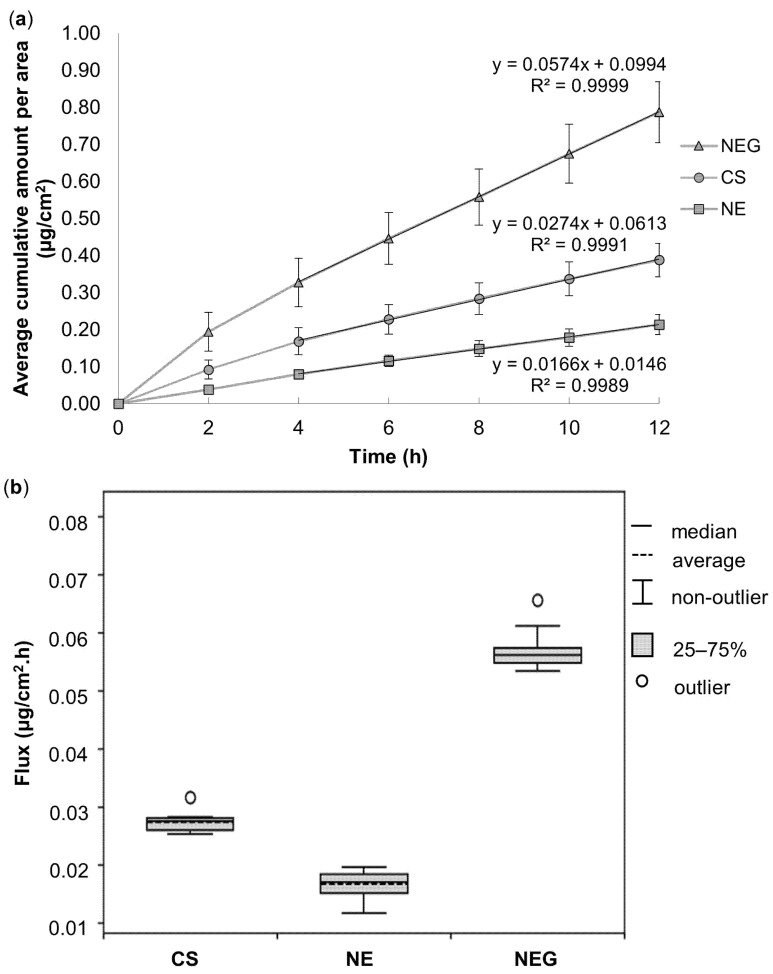
(**a**) Average cumulative amount of ivermectin diffused per area (μg/cm^2^) over 12 h during the ex vivo skin diffusion studies from all the nano-drug delivery vehicles, and (**b**) boxplot displaying the average (dashed lines) and median (solid lines) flux (μg/cm^2^.h) of ivermectin for the nano-drug delivery vehicles during the ex vivo skin diffusion studies over a period of 12 h.

Table 4 and Figure 4 represent the ex vivo skin diffusion data obtained for all three nano-drug delivery vehicles. The discussion of the results obtained for the nano-drug delivery vehicles will follow in terms of median %diffused as it takes the initial concentration into consideration and the median values are more accurate, being less affected by outliers compared to the average values [89].

The **CS**, interestingly, exhibited a higher median %diffused in comparison to the **NEG** and the **NE**, which displayed lower results; thus, surpassing expectations. Despite the **CS** containing only 0.35% ivermectin in comparison to **NE** and **NEG** containing 2.00% ivermectin, it displayed the highest ivermectin %diffused, confirming its vast drug delivery potential as drug delivery vehicle. **NPs** generally penetrate the skin through the follicular and intercellular route [97]. The follicular route has been well established for the transdermal delivery of **NPs** [97]. **NPs** are reportedly transported through the hair follicles, where they accumulate within the lower regions of the infundibulum, serving as a reservoir [48,98]. This leads to a depot effect and the controlled release of ivermectin. The hair follicle releases ivermectin into the systemic circulation, through the middle vascular plexus surrounding the hair follicles, as ivermectin is continuously made available from the saturated infundibulum [48]. Thus, explaining the successful transdermal delivery of ivermectin, despite the lower concentration thereof within the nano-drug delivery vehicle. Furthermore, the ivermectin-loaded **NPs** were suspended in EPO, a penetration enhancer that enters the lipid bilayers of the skin and disrupts their ordered domains, which may have contributed to the improved penetration of intact ivermectin-loaded **NPs** via the intercellular route of the skin [42,97].

Since the inclusion of a gelling agent is generally known to cause prolonged release of the API from its vehicle, it was unsuspected that the **NEG** would improve the transdermal permeation of ivermectin more than the **NE** [94]. It is worth noting that the **NEG** demonstrated the superlative rate of ivermectin delivery, exhibiting the highest flux through the skin in comparison to the other nano-drug delivery vehicles (**NE** and **CS**). **NEGs** are known to provide better skin adhesion and demonstrate increased solubilization capacity. This, subsequently, creates a larger concentration gradient across the skin and finally improves skin penetration [78,99]. Moreover, the occlusive effect of **NEGs** improves the hydration of the skin’s surface and reduces corneocyte packing within the stratum corneum, leading to improved API skin penetration [100,101].

It is worth mentioning that the amount of ivermectin that diffused from each nano-drug delivery vehicle (**NE**: 0.115 ± 0.014 μg/mL; **NEG**: 0.423 ± 0.044 μg/mL, and **CS**: 0.209 ± 0.024 μg/mL) surpassed the recorded therapeutic blood concentration, as described by Chhaiya et al. as 0.030–0.046 µg/mL [102]. These findings serve merely as a qualitative prediction of the potential therapeutic effect in subsequent in vivo studies and the API’s in vitro potency is of no concern, owing in part to the API’s pharmacokinetic features like skin metabolism, enzymatic degradation, elimination, etc., that emerge during in vivo testing [103,104].

A one-way ANOVA showed a statistically significant difference between the different nano-drug delivery vehicles’ flux values during the ex vivo skin diffusion study (*p* < 0.001); therefore, the data were further analyzed by means pair-wise Mann–Whitney U tests. All the nano-drug delivery vehicles indicated statistically significant differences (<0.05); therefore, these data were not generated at random but rather attributable to a specific cause [96].

### 3.5. Tape Stripping

**Figure 5:**
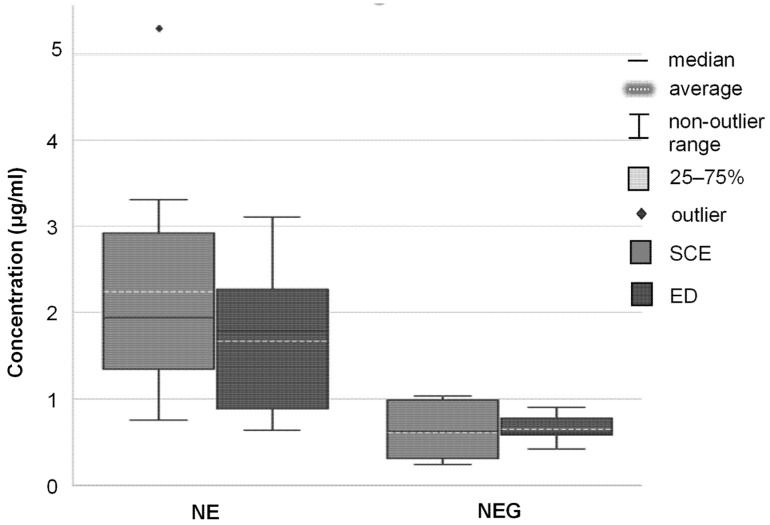
Boxplot displaying the average (dashed lines) and median (solid lines) concentration (μg/mL) of ivermectin from the different nano-drug delivery vehicles (**NE** and **NEG**) that were delivered in the SCE and ED after each 12 h ex vivo skin diffusion study.

The topical data obtained regarding the SCE and ED are presented in Table 5 and Figure 5 and will be discussed in terms of median values [89]. From the results presented in Table 5, it was established that ivermectin partitioned out of the **NE** and **NEG** and permeated into the SCE. The **NE** displayed the highest median concentration of ivermectin within the SCE, followed by **NEG**, while unquantifiable concentrations of ivermectin were retrieved from the **CS**.

Both the **NE** and **NEG** contained the same quantity of EPO, a penetration enhancer that acts by disrupting the packing order of the lipid bilayers and retaining the partitioned ivermectin within the SCE [42]. Furthermore, the transepidermal pathway (consisting of the transcellular and intercellular pathways) is widely considered the predominant pathway of skin permeation [105]. As ivermectin is a lipophilic molecule, it was most likely transported via the intercellular pathways by diffusing through the continuous lipid matrix present between the cells [106]. When comparing the results obtained for both **NE** and **NEG**, the **NE** presented with the higher concentration of ivermectin retrieved from the SCE of the two nano-drug delivery vehicles. As the only difference between the two nano-drug delivery vehicles is the addition of a gelling-agent (Carbopol^®^ Ultrez 20) to the **NEG**, it can likely be ascribed to a film-forming effect that is usually seen with gelling agents. This, subsequently, results in prolonged API permeation through the skin [97,107].

The retrieval of ivermectin from the ED, despite the API’s expected affinity to the SCE, is an indication that the lipid barrier of the stratum corneum was overcome and that the lipophilic ivermectin permeated to the deeper, hydrophilic layers of the skin [107]. The **NE** measured the highest concentration ivermectin retrieved from the ED, followed by the **NEG**. Once again, the concentrations of ivermectin retrieved from the ED treated with the **CS** was unquantifiable. The concentrations of ivermectin retrieved from the ED for both the **NE** and **NEG** correlate with the concentrations retrieved from the SCE, respectively. Roohnikan et al. [108] explains that the concentration of the API observed within the SCE could increase the concentration of the API within the ED, as a concentration gradient is created across the layers, acting as a driving force. When comparing the **NE** and **NEG** to one another, a higher concentration of ivermectin was retrieved in the ED for the **NE**, once again attributed to the addition of a gelling agent to **NEG**, causing an overall prolonged API permeation [109]. Another factor to take into consideration is the larger droplet size measured for **NEG** (when compared to **NE**), which could also have decreased permeation into the stratum corneum and further influence permeation into the deeper layers of the skin.

The tape stripping data obtained from the two groups (**NE** and **NEG**) were statistically analyzed in terms of mean ivermectin concentrations found in the SCE and the ED, respectively, and therefore, a two-way ANOVA was employed to determine the statistically significant differences. No statistically significant interactions were reported; thus, the main effects were analyzed for further interpretation. The conclusion was drawn that the different nano-drug delivery vehicles (**NE** and **NEG**) had the largest effect on the concentration of ivermectin retained in the different layers of the skin and was the only factor of statistical significance. The layers of the skin, as well as the interaction between the skin layer and the nano-drug delivery vehicles, had no statistically significant effect on the retention of ivermectin. The Mann–Whitney post hoc U-test was utilized to compare the mean concentrations of ivermectin from each drug delivery vehicle (excluding the skin layer, i.e., SCE and ED). Statistically significant differences were observed between **NE** and **NEG**, as *p* < 0.05. It was therefore concluded that the nano-drug delivery vehicles affected the data and were not a random circumstance [96].

### 3.6. In Vitro Cytotoxicity Studies

**Figure 6:**
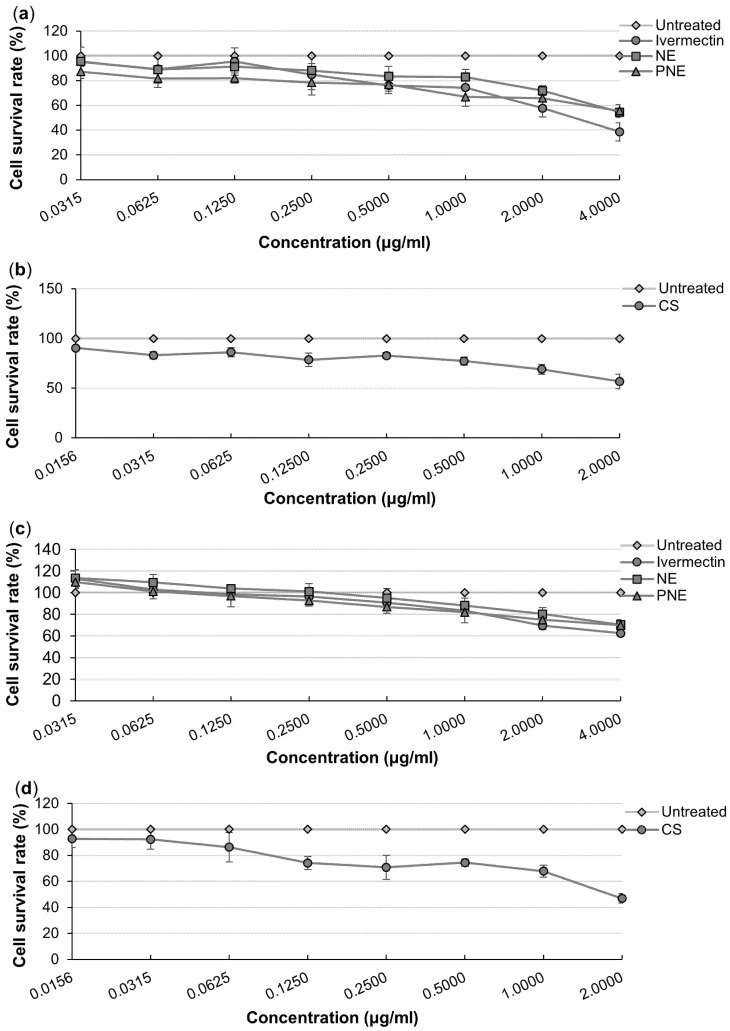
%Cell viability of the HaCaT cells after treatment with different concentrations of (**a**) API, **NE** and **PNE**, and (**b**) **CS** using the MTT assay, while the NR assay was used for (**c**) API, **NE** and **PNE**, and (**d**) **CS**.

**Figure 7:**
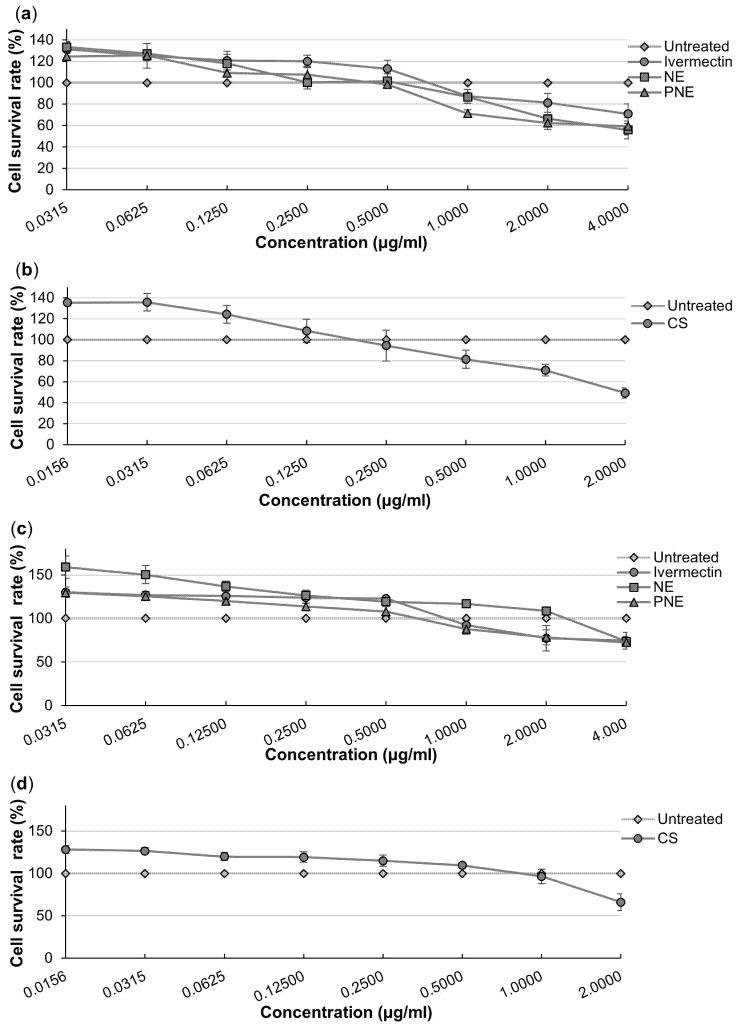
%Cell viability of the BJ-5sta cells after treatment with different concentrations of (**a**) API, **NE** and **PNE**, and (**b**) **CS** using the MTT assay, while the NR assay was used for (**c**) API, **NE** and **PNE**, and (**d**) **CS**.

The cell viability of the cultures was calculated relative to the untreated cell control, indicated as 100% viability [110]. Figure 6 (HaCaT cells) and Figure 7 (BJ-5sta cells) present the results expressed as graphs for the MTT and NR assays.

#### 3.6.1. Methyl Thiazolyl Tetrazolium Assay

No cytotoxicity (>80% cell viability) was reported for most of the concentrations when interpreting the results obtained for both cell lines (HaCaT and BJ-5ta). For both cell lines (HaCaT and BJ-5ta) treated with the **NE**, no cytotoxicity was reported up to a concentration of 1.0000 μg/mL. Weak (60–80% cell viability) and moderate (40–60% cell viability) cytotoxicity were observed during the treatment of both cell lines with **NE** at 2.0000 and 4.0000 μg/mL, respectively. **PNE** treatment of the HaCaT cells presented no cytotoxicity up to 0.1250 μg/mL, followed by weak cytotoxicity between 0.2500 and 2.0000 μg/mL and moderate cytotoxicity at 4.0000 μg/mL. Treatment of the BJ-5ta presented no cytotoxicity for concentrations up to 0.5000 μg/mL, while weak cytotoxicity was observed between 1.0000 μg/mL and 2.0000 μg/mL for the **PNE** treatment. Moderate cytotoxicity was observed in the BJ-5ta cells treated with 4.0000 μg/mL of **PNE**. The **CS** treatment on HaCaT cells exhibited no cytotoxicity within the concentrations of 0.0156–0.6250 μg/mL and at 0.2500 μg/mL. Weak cytotoxicity was observed at 0.1250, 0.5000 and 1.0000 μg/mL, with moderate cytotoxicity at 2.0000 μg/mL in the treatment of HaCaT cells with **CS**. No cytotoxicity was observed in the treatment of BJ-5ta cells within most of the concentration range of **CS**, except weak and moderate cytotoxicity which occurred at 1.0000 μg/mL and 2.0000 μg/mL, respectively [110].

#### 3.6.2. Neutral Red Assay

It was observed that the HaCaT and BJ-5ta cells treated with the API (ivermectin) showed no toxicity at concentrations of 0.0315–1.0000 μg/mL, as %cell viability was above 80%. Weak cytotoxicity (60–80% of cell viability) was observed in both HaCaT and BJ-5ta cell lines at 2.0000 and 4.0000 μg/mL of ivermectin treatment. The same was true for the **PNE** treatment with both cell lines, where only weak cytotoxicity was reported at 2.0000 and 4.0000 μg/mL of **PNE** treatment. The **NE** treatment presented with no cytotoxicity throughout most of the concentration ranges apart from weak cytotoxicity occurring at 4.000 μg/mL in both cell lines. HaCaT cells treated with **CS** presented with no cytotoxicity up to 0.0625 μg/mL, weak cytotoxicity between 0.1250 and 1.0000 μg/mL and moderate cytotoxicity (40–60% of cell viability) at 2.0000 μg/mL. With regard to **CS** treatment on BJ-5ta cells, no cytotoxicity was reported for the entire concentration range with weak cytotoxicity observed only at 2.0000 μg/mL [110].

## 4. Conclusions

From the solubility studies, it was established that ivermectin is more lipophilic in nature and exhibits extremely poor aqueous solubility, which is in accordance with the literature [36,37]. When referring to the data obtained from the in vitro membrane release studies, ivermectin was successfully released from all three nano-drug delivery vehicles (**NE**, **NEG** and **CS**). This was followed by ex vivo skin diffusion studies and tape stripping that demonstrated the successful delivery of all three nano-drug delivery vehicles through the skin, with the **NE** and **NEG** successfully penetrating the skin (SCE and ED). The **NE** produced sub-par results with regard to the transdermal drug delivery of ivermectin. It did, however, demonstrate the best results of all the nano-drug delivery vehicles regarding release from the drug delivery vehicles and topical drug delivery with the highest concentration of ivermectin present in both the SCE and ED. The **NE**, thus, exhibits great potential for the development of a topical application of ivermectin as the API penetrated and was retained within the deeper layers of the skin. The **NEG** demonstrated an accelerated delivery rate of ivermectin compared to the other nano-drug delivery vehicles (**NE** and **CS**), with the second highest %ivermectin diffused and the second highest concentration of ivermectin within both skin layers (SCE and ED). The improved skin adhesion and solubilization capacity, as well as the occlusive properties presented by the **NEG**, enabled the API to successfully penetrate the skin and created an increased concentration gradient across the deeper layers of the skin, serving as a driving force for the diffusion of ivermectin from the SCE, through the ED and into systemic circulation [48,78,97,108]. When interpreting the results obtained from the **CS**, it is noteworthy that it only contained 0.35% of the API in comparison with the other nano-drug-delivery vehicles, containing 2.00% ivermectin. The **CS** presented with the highest %ivermectin diffused, highlighting the promise it possesses as a transdermal drug delivery vehicle. Additionally, undetectable concentrations of ivermectin were recovered from both the SCE and ED, confirming that the follicular pathway was the primary route of delivering ivermectin transdermally [97,111].

In conclusion, the type of nano-drug delivery vehicle in which ivermectin is incorporated affects the delivery thereof (topically and transdermally), demonstrating the continuous change in equilibrium between the API, vehicle and skin [112,113].

Finally, no cytotoxicity and/or weak cytotoxicity was observed from most of the API, **NE**, **PNE** and **CS** treatment concentrations during both MTT and NR assays; however, some higher degrees of cytotoxicity were observed from the API treatments (MTT assay of HaCaT cells) with moderate and strong cytotoxicity at the higher end of the concentration range [110]. These in vitro cytotoxicity studies are, however, not precise indicators for in vivo studies, lacking pharmacokinetic properties such as absorption, metabolism and excretion [114].

Future research could explore incorporating various solid-state forms of ivermectin by modifying its crystal structure, aiming to enhance its physicochemical properties and evaluate the potential for improved transdermal and topical drug delivery. Enhanced physicochemical properties are expected to increase bioavailability, potentially enabling lower-dose administration with heightened therapeutic efficacy and reduced side effects [115]. Additionally, investigating natural oils rich in skin-compatible fatty acids may provide safer alternatives to conventional chemical penetration enhancers while also enhancing skin permeation [45,46]. Other nano-drug delivery systems should also be explored to optimize delivery outcomes. Once an optimal combination of nano-drug delivery vehicle, solid-state form and penetration enhancer is identified, in vivo testing can be initiated.

## Figures and Tables

**Table 1:** Formula used to produce the applicable nano-drug delivery vehicles.

Phase	Excipient	Nano-Drug Delivery Vehicle (%*w*/*v*)			
		NE	NEG	NP	CS
**Oil phase**	EPO	20.000	20.000	-	-
	Ivermectin	2.000	2.000	-	-
	Span^®^ 60	2.400	2.400	-	-
**Aqueous phase**	UP Water	66.000	65.300	90.450	-
	Tween^®^ 80	9.600	9.600	-	-
	Carbopol^®^ Ultrez 20	-	0.700	-	-
	PVA	-	-	0.451	-
**Organic phase**	DCM	-	-	9.045	-
	Ivermectin	-	-	0.027	-
	PCL	-	-	0.027	-
**Solid**	Ivermectin-loaded **NPs**	-	-	-	0.900

EPO—evening primrose oil; UP water—ultrapure water; PVA—polyvinyl alcohol; DCM—dichloromethane; PCL—polycaprolactone, **NPs**—nanoparticles; **NE**—nano-emulsion; **NEG**—nano-emulgel; **CS**—colloidal suspension.

**Table 2:** Summarized characterization results for each nano-drug delivery vehicle.

	NE	NEG	NPs	CS
**pH**	5.548 ± 0.004	6.201 ± 0.006	-	4.226 ± 0.012
**Viscosity (cP)**	25.9 ± 0.6	16,858.0 ± 237.8	-	-
**Droplet/particle size (nm)**	57.157 ± 0.455	106.900 ± 0.490	173.800 ± 18.400	-
**PDI**	0.165 ± 0.014	0.287 ± 0.037	0.359 ± 0.035	-
**Zeta-potential (mV)**	−30.600 ± 1.300	−40.400 ± 1.283	-	−36.200 ± 0.666

**PDI**—polydispersity index; **NE**—nano-emulsion; **NEG**—nano-emulgel; **NPs**—nanoparticles; **CS**—colloidal suspension.

**Table 3:** In vitro membrane release data for each nano-drug delivery vehicle (*n* = number of Franz cells used).

Nano-Drug Delivery Vehicle	*n*	Average %Release (%)	Median %Release (%)	Average Flux (μg/cm^2^.h)	Median Flux (μg/cm^2^.h)
**NE**	9	2.003 ± 0.342	1.979	611.155 ± 96.820	608.550
**NEG**	8	1.500 ± 0.206	1.569	489.770 ± 76.930	507.705
**CS**	10	0.252 ± 0.009	0.248	2.346 ± 0.119	2.326

**NE**—nano-emulsion; **NEG**—nano-emulgel; **CS**—colloidal suspension.

**Table 4:** Ex vivo skin diffusion data for each nano-drug delivery vehicle (*n* = number of Franz cells used).

Nano-Drug Delivery Vehicle	*n*	Average %Diffused (%)	Median %Diffused (%)	Average Flux (μg/cm^2^.h)	Median Flux (μg/cm^2^.h)
**NE**	9	0.001 ± 0.000	0.001	0.017 ± 0.003	0.017
**NEG**	9	0.002 ± 0.000	0.002	0.057 ± 0.004	0.056
**CS**	10	0.006 ± 0.001	0.006	0.027 ± 0.002	0.028

**NE**—nano-emulsion; **NEG**—nano-emulgel; **CS**—colloidal suspension.

**Table 5:** Tape stripping data (SCE and ED) for each nano-drug delivery vehicle (*n* = number of skin samples).

Nano-Drug Delivery Vehicle	*n*	Average Concentration in SCE (μg/mL)	Median Concentration in SCE (μg/mL)	Average Concentration in ED (μg/mL)	Median Concentration in ED (μg/mL)
**NE**	9	2.241 ± 1.425	1.935	1.662 ± 0.886	1.786
**NEG**	9	0.610 ± 0.323	0.614	0.648 ± 0.164	0.605
**CS**	10	0.000 ± 0.000	0.000	0.000 ± 0.000	0.000

**NE**—nano-emulsion; **NEG**—nano-emulgel; **CS**—colloidal suspension; SCE—stratum corneum-epidermis; ED—epidermis dermis.

## Data Availability

The datasets generated and/or analyzed during the current research project are available from the corresponding author upon reasonable request.

## Supplementary Materials

The following supporting information can be downloaded at: https://www.mdpi.com/article/10.3390/pharmaceutics16111466/s1, Table S1: Formulas used for the four various **NEs**; Table S2: Summary of the characterization results for all four pre-formulated o/w **NEs**; Table S3: Entrapment efficiency (%) and unentrapped (free) percentage of ivermectin within the respective pre-formulated o/w **NEs**; Table S4: Formulas used for the four various **NEGs**; Table S5: Summary of the characterization results for all four pre-formulated o/w **NEGs**. References [116,117,118,119,120] are cited in the supplementary materials.
