# Supplementary material for: Formulation and Ex Vivo Evaluation of Ivermectin Within Different Nano-Drug Delivery Vehicles for Transdermal Drug Delivery
Source: Pharmaceutics. 2024 Nov 18;16(11):1466. doi: 10.3390/pharmaceutics16111466 (PMC11597838; doi:10.3390/pharmaceutics16111466)
Supplement: Supplementary file 1 [file pharmaceutics-16-01466-s001.zip › pharmaceutics-3287359-supplementary.pdf]

Supplementary material

Formulation and *ex vivo* evaluation of ivermectin within different nano-drug delivery vehicles for transdermal drug delivery

Eunice Maureen Steenekamp [1], Wilna Liebenberg [1], Hendrik J R Lemmer [1] and Minja Gerber [1,*]

[1]Centre of Excellence for Pharmaceutical Sciences (Pharmacen™), Faculty of Health Sciences, North-West University, Private Bag X6001, Potchefstroom, 2520, South Africa; eunicesteenekamp13@gmail.com (E.M.S.); wilna.liebenberg@nwu.ac.za (W.L.); righard.lemmer@nwu.ac.za (H.J.R.L.).

*Correspondence: minja.gerber@nwu.ac.za (M.G.); Tel.: +27 18 299 2328

Table of Contents

Composition of nano-emulsion formulae 1

Preformulation characterization results of the nano-emulsions 2

Composition of nano-emulgel formulae 4

Preformulation characterization results of the nano-emulgels 5

Composition of nano-emulsion formulae

All four nano-emulsions (**NE1–NE4**) contained the same amount of oil phase (evening primrose oil (EPO) at 20%). The total surfactant concentration was 10% for **NE1** and **NE2** and 12% for **NE3** and **NE4**. The Span® 60: Tween® 80 ratios for **NE1–NE4** were 1:6, 1:4, 1:4, and 1:2, respectively.

Table S1. Formulas used for the four various **NEs**

	Phase	Excipients	Quantity (g; %*w*/*w*)
**NE1**	Oil phase	EPO	10.000 g (20.00%)
		Ivermectin	1.000 g (2.00%)
		Span® 60	1.000 g (2.00%)
	Aqueous phase	Tween® 80	4.000 g (8.00%)
		UP water	34.000 g (68.00%)
**NE2**	Oil phase	EPO	10.000 g (20.00%)
		Ivermectin	1.000 g (2.00%)
		Span® 60	0.715 g (1.43%)
	Aqueous phase	Tween® 80	4.285 g (8.57%)
		UP water	34.000 g (68.00%)
**NE3**	Oil phase	EPO	10.000 g (20.00%)
		Ivermectin	1.000 g (2.00%)
		Span® 60	1.200 g (2.40%)
	Aqueous phase	Tween® 80	4.800 g (9.60%)
		UP water	33.000 g (66.00%)
**NE4**	Oil phase	EPO	10.000 g (20.00%)
		Ivermectin	1.000 g (2.00%)
		Span® 60	2.000 g (4.00%)
	Aqueous phase	Tween® 80	4.000 g (8.00%)
		UP water	33.000 g (66.00%)

Preformulation characterization results of the nano-emulsions

Table S2. Summary of the characterization results for all four pre-formulated o/w **NEs**

	**pH**	**Droplet size (nm)**	**PDI**	**Zeta-potential (mV)**	**Viscosity (cP)**
**NE1**	5.508±0.008	167.233±0.998	0.141±0.026	-27.2±1.4	56.6±1.4
**NE2**	5.385±0.004	104.567±2.167	0.201±0.018	-26.6±0.8	107.3±1.3
**NE3**	5.548±0.004	57.157±0.455	0.165±0.014	-30.6±1.3	25.9±0.6
**NE4**	5.490±0.004	69.633±0.957	0.207±0.007	-29.0±0.2	56.5±1.3

From Table S2, it can be observed that **NE3** and **NE4** exhibited smaller droplet sizes than **NE1** and **NE2**. This difference is likely due to **NE3** and **NE4** containing a total surfactant concentration of 12%, while **NE1** and **NE2** contained a lower total surfactant concentration of 10%. Chang et al. [116] found that higher surfactant concentrations in a nano-emulsion are associated with smaller droplets.

Nano-emulsions are known to have limited drug-loading capacity. Therefore, achieving a 2% (w/w; 1 000 mg of ivermectin in 50 ml) concentration fully entrapped within the oil phase of these nano-emulsions would likely be challenging without significantly increasing the oil phase, which is already at 20%. Research conducted by An et al. [117] indicated that larger droplet sizes are formed when increasing the oil phase, potentially resulting in instability, including flocculation, creaming and sedimentation [118]. Furthermore, larger droplets could reduce the effectiveness of transdermal delivery, since nanosized droplets are vital for enhancing skin permeation [119]. Therefore, incorporating a combination of lipophilic surfactants, like Span® 60, and hydrophilic surfactants, such as Tween® 80, can improve entrapment efficacy. Lipophilic surfactants enhance entrapment in the oil phase, while hydrophilic surfactants improve solubility of the active pharmaceutical ingredient (API) in the aqueous phase [120], which alleviates instability, since no aggregation or sedimentation was observed, while maintaining a homogenous (low PDI) formulation across all four nano-emulsions.

Despite the relatively low entrapment efficiency observed in Table S3, the high concentration of surfactants (10–12% w/w) stabilized the nano-sized droplets and improved API solubility in the water phase. This enabled the inclusion of a high percentage free ivermectin in the nano-emulsions (**NE1**–**NE4**) without compromising stability or homogeneity, allowing nearly the entire 2% (w/w) of ivermectin to be incorporated in the nano-emulsion,

which is likely due to the high (and favorable) Tween® 80 to Span® 60 ratio.

Table S3. Entrapment efficiency (%) and unentrapped (free) percentage of ivermectin within the respective pre-formulated o/w **NEs**

	**Entrapment efficiency (%)**	**Unentrapped percentage (%)**
**NE1**	17.87	81.39
**NE2**	17.88	81.22
**NE3**	18.36	80.98
**NE4**	18.11	81.12

Composition of nano-emulgel formulae

Table S4. Formulas used for the four various **NEGs**

	**Phase**	**Excipients**	**Quantity (g; %*w*/*w*)**
**NEG1**	Oil phase	EPO	10.00 g (20.0%)
		Ivermectin	1.00 g (2.0%)
		Span® 60	1.20 g (2.4%)
	Aqueous phase	Carbopol® Ultrez 20	0.35 g (0.7%)
		Tween® 80	4.80 g (9.6%)
		UP water	32.65 g (65.3%)
**NEG2**	Oil phase	EPO	10.00 g (20.0%)
		Ivermectin	1.00 g (2.0%)
		Span® 60	1.20 g (2.4%)
	Aqueous phase	Carbopol® Ultrez 20	0.40 g (0.8%)
		Tween® 80	4.80 g (9.6%)
		UP water	32.60 g (65.2%)
**NEG3**	Oil phase	EPO	10.00 g (20.0%)
		Ivermectin	1.00 g (2.0%)
		Span® 60	1.20 g (2.4%)
	Aqueous phase	Carbopol® Ultrez 20	0.45 g (0.9%)
		Tween® 80	4.80 g (9.6%)
		UP water	32.55 g (65.1%)
**NEG4**	Oil phase	EPO	10.00 g (20.0%)
		Ivermectin	1.00 g (2.0%)
		Span® 60	1.20 g (2.4%)
	Aqueous phase	Carbopol® Ultrez 20	0.30 g (0.6%)
		Tween® 80	4.80 g (9.6%)
		UP water	32.70 g (65.4%)

Preformulation characterization results of the nano-emulgels

Table S5. Summary of the characterization results for all four pre-formulated o/w **NEGs**

	pH	Droplet size (nm)	PDI	Zeta-potential (mV)	Viscosity (cP)
**NEG1**	6.201±0.006	106.900±0.490	0.287±0.037	-40.4±1.3	16,858±237.8
**NEG2**	6.221±0.009	116.100±1.883	0.354±0.026	-47.3±1.1	19,098±57.4
**NEG3**	6.256±0.006	137.433±1.901	0.285±0.019	-48.4±0.9	24,740±130.2
**NEG4**	6.259±0.014	129.233±1.488	0.272±0.018	-32.7±0.2	16,548±51.1
